# Supplementary material for: LSEA Evaluation of Lipid Mediators of Inflammation in Lung and Cortex of Mice Exposed to Diesel Air Pollution
Source: Biomedicines. 2022 Mar 19;10(3):712. doi: 10.3390/biomedicines10030712 (PMC8945792; doi:10.3390/biomedicines10030712)
Supplement: Supplementary file 1 [file biomedicines-10-00712-s001.zip › Supplemental Tables.pdf]

Supplemental Table S1. Chemical compositions of UFPs from DEP. Each value is expressed as mean concentrations (±SD)

Table S1 Chemical compositions of DEP, reported in Longhin et al., 2016. [15] Each value is expressed as mean concentrations (± SD).

Elements	Unit	DEP
Al	ng/µg	135 ±4
K	ng/µg	50 ±0.02
Ca	ng/µg	198 ±8
Fe	ng/µg	4 ±0.001
Zn	ng/µg	70 ±2
Cr	ng/µg	0.04 ±0.001
Mn	ng/µg	0.03 ±0.001
V	ng/µg	0.05 ±0.007
Ni	ng/µg	0.02 ±0.001
Pb	ng/µg	0.02 ±0.001
Total PAHs	ng/mg	600 ±150

Supplemental Table S2. Lipid mediator metadata, raw and normalized data

Supplemental Table S3. Differential lipid mediator enrichment results

Supplemental Table S4. LSEA functional enrichment results

Supplemental Table S5. Multivariate analysis results
